# Supplementary material for: Hybrid Models and Biological Model Reduction with PyDSTool
Source: PLoS Comput Biol. 2012 Aug 9;8(8):e1002628. doi: 10.1371/journal.pcbi.1002628 (PMC3415397; doi:10.1371/journal.pcbi.1002628)
Supplement: Text S4 — Complete source code for the PyDSTool package (version 0.88.120504). Includes API documentation and help files linking to web pages. This file is identical to the current public release on Sourceforge.net. (ZIP) [file pcbi.1002628.s004.zip › PyDSTool/html/PyDSTool.Toolbox.neuro_data.spike_envelope-class.html]

xml version="1.0" encoding="ascii"?


PyDSTool.Toolbox.neuro\_data.spike\_envelope


| Home | Trees | Indices | Help | PyDSTool |
| --- | --- | --- | --- | --- |

Package PyDSTool ::
Package Toolbox ::
Module neuro\_data ::
Class spike\_envelope

[hide private]

[frames] | no frames]

# Class spike\_envelope

source code

```
object --+
         |
        spike_envelope
```

---

Find an amplitude envelope over a smooth 1D signal that features
roughly periodic spikes. Input is a 1D parameterized pointset and the
approximate period. An optional input is the tolerance (fraction) for
finding spikes around the period (measuring uncertainty in the period) --
default 0.2 (20% of the period).

Optional start\_t sets where to orient the search in the independent
variable -- default None (start at the highest point of the signal). It
\*must\* be a value that is present in the independent variable array of
the given points argument.

Optional noise\_floor sets minimum signal amplitude considered to be a
peak (default 0 means non-noisy data assumed).

Outside of spike times +/- tol, envelope curve will be defined as
amplitude zero.

adjust\_rate is a fraction < 1 specifying the %age change of spike
search interval (a.k.a. 'period'). default 0.1.

make\_traj option can be used to avoid costly creation of a Trajectory
object representing the envelope curve, if unneeded (default True).

When less is known in advance about the regularity or other properties
of the spikes, pre-process using estimate\_spiking() and pass the result
as the optional argument spest.


|  |  |
| --- | --- |
| Instance Methods | [hide private] |

|  |  |
| --- | --- |
| \_\_init\_\_(self, pts, per, tol=0.2, start\_t=None, noise\_floor=0, depvar=None, adjust\_rate=0.1, make\_traj=True, spest=None)  x.\_\_init\_\_(...) initializes x; see x.\_\_class\_\_.\_\_doc\_\_ for signature | source code |

|  |  |
| --- | --- |
| find\_spike\_ixs\_dir(self, dir=1, per=None, start\_t=None, tol=None)  Use dir=-1 for backwards direction | source code |

**Inherited from `object`**:
`__delattr__`,
`__getattribute__`,
`__hash__`,
`__new__`,
`__reduce__`,
`__reduce_ex__`,
`__repr__`,
`__setattr__`,
`__str__`


|  |  |
| --- | --- |
| Properties | [hide private] |

**Inherited from `object`**:
`__class__`


|  |  |
| --- | --- |
| Method Details | [hide private] |

|  |  |
| --- | --- |
| \_\_init\_\_(self, pts, per, tol=0.2, start\_t=None, noise\_floor=0, depvar=None, adjust\_rate=0.1, make\_traj=True, spest=None)  *(Constructor)* | source code |

x.\_\_init\_\_(...) initializes x; see x.\_\_class\_\_.\_\_doc\_\_ for
signature

Overrides: object.\_\_init\_\_: *(inherited documentation)*

  


| Home | Trees | Indices | Help | PyDSTool |
| --- | --- | --- | --- | --- |

|  |  |
| --- | --- |
| Generated by Epydoc 3.0.1 on Fri May 4 15:24:09 2012 | http://epydoc.sourceforge.net |
